# Systemic administration of a broadly-neutralizing IgG antibody to generate HIV-neutralization responses in breast milk

**DOI:** 10.1186/1742-4690-9-S2-P196

**Published:** 2012-09-13

**Authors:** GG Fouda, J Amos, K Beck, S Smith, X Wang, K Reimann, S Permar

**Affiliations:** 1 Duke Human Vaccine Institute, Durham, NC, USA; 2 Beth Israel Deaconess Medical Center, Boston, MA, USA

## Background

Postnatal acquisition of HIV during breastfeeding is responsible for almost half of 350,000 pediatric HIV infections occurring yearly. Thus, there is an urgent need to develop immunologic interventions to impede breast milk transmission of HIV, including immunization and passive infusion strategies. We previously observed that functional antibody responses in milk of HIV- infected mothers mirror that in plasma, suggesting that inducing strong systemic IgG responses may lead to virus inhibition in milk. Therefore, we investigated the kinetics of binding and neutralizing antibodies in plasma and milk of passively- infused lactating rhesus monkeys.

## Methods

The broadly neutralizing antibody b12 engineered in a rhesus IgG1 backbone was administered intravenously to four hormone-induced, lactating female rhesus monkeys at a dose of 5mg/kg. Milk and blood was collected frequently until 72 h post infusion, then weekly for 4 weeks. Levels of the infused antibody and the neutralizing activity in the milk and systemic compartments were measured at each time-point.

## Results

The b12 IgG levels peaked 1 hour post-infusion in plasma and 24 to 72h post-infusion in milk. The median peak b12 antibody levels were 87,503 ng/ml (range 62,548 to 101,525 ng/ml) in plasma and 47 ng/ml (range 16 to 202 ng/ml) in milk. The peak in plasma neutralization was 1 to 6 hours post-infusion and the neutralization titer slowly declined after 24 hours. The peak neutralization titer in milk (median ID50: 70, range: 50-103) was approximately two logs lower than in plasma (median ID50: 2313, range: 1875-3128) and occurred within 24 hours post-infusion in 3 of 4 animals. There was a significant correlation between neutralization titers in milk and plasma (r=0.48, p=0.01).

## Conclusion

The neutralizing activity detected in milk following systemic administration of a broadly- neutralizing IgG antibody supports the induction of strong systemic anti-HIV IgG responses to generate HIV inhibitory antibodies in breast milk.

